# Evidence for Involvement of IL-9 and IL-22 in Cows’ Milk Allergy in Infants

**DOI:** 10.3390/nu9101048

**Published:** 2017-09-21

**Authors:** Karina V. Barros, Vera L. Flor Silveira, Marisa S. Laranjeira, Neusa F. Wandalsen, Susana Passeti, Roberta de Oliveira, Regina V. Munekata, Paul S. Noakes, Elizabeth A. Miles, Philip C. Calder

**Affiliations:** 1 Department of Physiology, Federal University of São Paulo, São Paulo SP CEP 04023-900, Brazil; karinavbarros@hotmail.com; 2 Department of Biological Sciences, Federal University of São Paulo, Diadema SP CEP 09972-270, Brazil; veraflorsilveira@gmail.com; 3 Department of Pediatrics, Faculty of Medicine, Federal University of ABC, Santo André SP CEP 09210-580, Brazil; marisalaranjeira@uol.com.br (M.S.L.); nfwandalsen@uol.com.br (N.F.W.); susanapasseti@yahoo.com.br (S.P.); robs38@bol.com.br (R.d.O.); rvmunekata@hotmail.com (R.V.M.); 4 Human Development and Health Academic Unit, Faculty of Medicine, University of Southampton, Southampton SO16 6YD, UK; paul.noakes@nd.edu.au (P.S.N.); eam@soton.ac.uk (E.A.M.); 5 National Institute for Health Research Southampton Biomedical Research Centre, University Hospital Southampton NHS Foundation Trust and University of Southampton, Southampton SO16 6YD, UK

**Keywords:** cows’ milk allergy, cytokine, dietetic treatment, hydrolysed soy formula, free amino acid formula

## Abstract

Although allergic inflammation is characterized by a T helper (Th) 2-dominant immune response, the discovery of a role for new T cell subsets in inflammatory diseases has added an additional layer of complexity to the understanding of the pathogeneses of allergic diseases. We evaluated plasma cytokine profiles in infants with cows’ milk allergy (CMA), who were being treated with an elimination diet. In a prospective, randomized and controlled study, infants (aged 8.4 ± 3.9 months) with CMA were treated with an elimination diet for 120 days, which replaced cows’ milk with a hydrolysed soy protein formula (*n* = 26) or a free amino acid formula (*n* = 20). Blood samples were collected before treatment during active disease (T0) and after 120 days, when symptoms were absent (T1). Plasma cytokine concentrations were measured. Infants with CMA had higher plasma concentrations of interleukin (IL)-4 and IL-13 and lower concentrations of IL-9, IL-17A and interferon-γ, compared with healthy breast-fed infants. At T0, there was a positive correlation between blood eosinophil numbers and plasma concentrations of IL-4, IL-9, IL-17A and IL-22. Treatment with a cows’ milk elimination diet resulted in a decrease in plasma IL-4, IL-9, IL-13 and IL-22 and an increase in plasma IL-17A. We conclude that IL-4 and IL-13 are elevated in active CMA. The association of IL-9 and IL-22 with eosinophilia, and the decrease in these two cytokines with cows’ milk elimination, suggests that they both play a role in the symptoms observed in CMA and may be important targets for future interventions.

## 1. Introduction

Cows’ milk allergy (CMA) is the most common food allergy, overall and in infants [1]. A pan-European study identified a self-reported lifetime prevalence of CMA, across all ages, of 6%, a point prevalence of self-reported CMA of 2.3% and a prevalence of food challenge-defined CMA of 0.6% [1]. Another pan-European study reported an overall incidence of challenge-proven CMA of 0.54% at 2 years of age, but this varied between <0.3% and 1% among the different countries included [2]. CMA may be immunoglobulin (IgE)-mediated or non-IgE-mediated [3]. These two forms can produce different symptoms [3]. As an allergic disease, CMA involves dysregulation of the immune response to one or more cows’ milk (CM) proteins, and is commonly associated with chronic inflammation, characterized by the infiltration and accumulation of eosinophils, T cells and mast cells [4]. Classically, allergic inflammation is characterized by an initial T-helper (Th) 2 cell response (mediated by interleukin (IL)-4, IL-5 and IL-13) [5,6]. However, the dichotomy between Th1 and Th2 cells has been modified by the discovery of other T lymphocyte subsets, namely Th17 and Th22, which appear to play a role in the maintenance of inflammation in allergic diseases, when the disease progresses into a chronic phase [6,7,8]. Although the majority of infants recover from CMA by the age of 3 years, they are at an increased risk of developing atopic diseases, such as asthma, atopic dermatitis and rhinoconjunctivitis. In particular, those who are positive to skin prick testing (SPT), indicating increased production of allergen specific IgE, are at risk [9,10,11].

IL-17 and IL-22 are leukocyte-derived cytokines which impact inflamed tissues and epithelial cells [12]. Th17 cells—characterized by IL-17A, IL-17F, IL-6, tumor necrosis factor (TNF)-α, IL-21 and granulocyte–macrophage colony stimulating factor production—have been described in many inflammatory conditions, including arthritis, psoriasis, inflammatory bowel disease and eosinophilic airway inflammation as being responsible for inducing other pro-inflammatory cytokines and chemokines, such as IL-8 and IL-6 [12,13]. Th17 cells are associated with neutrophil and/or macrophage recruitment to sites of inflammation [12,13]. Th22 cells are characterized by the production of IL-22, which acts exclusively on non-hematopoietic IL-22 receptor-expressing cells in the skin, pancreas, intestine, liver, lungs and kidneys [12,14]. IL-22 has both pro-inflammatory and protective properties, and its role is not fully understood [12,14]. IL-9 was initially thought to be a Th2-specific cytokine, exerting effects on asthma pathogenesis, IgE class switching and the resolution of parasitic infection [15,16]. However, upon activation by antigen-presenting cells, in the presence of transforming growth factor β and IL-4, naïve cluster of differentiation 4^+^ T cells differentiate into Th9 cells, which release IL-10—although no regulatory effect is demonstrated after IL-10 production [16]. Nevertheless, because of this action, transforming growth factor β has been a focus of therapeutic strategies for allergic disease treatment [17].

The aim of this study was to evaluate the association of IL-9, IL-17 and IL-22 with CMA in infants. Treatment of the infants was done with an elimination diet using two alternatives to CM: hydrolysed soy protein formula (HSF) and free amino acid formula (AAF).

## 2. Materials and Methods 

### 2.1. Participants and Study Design

**Figure 1:**
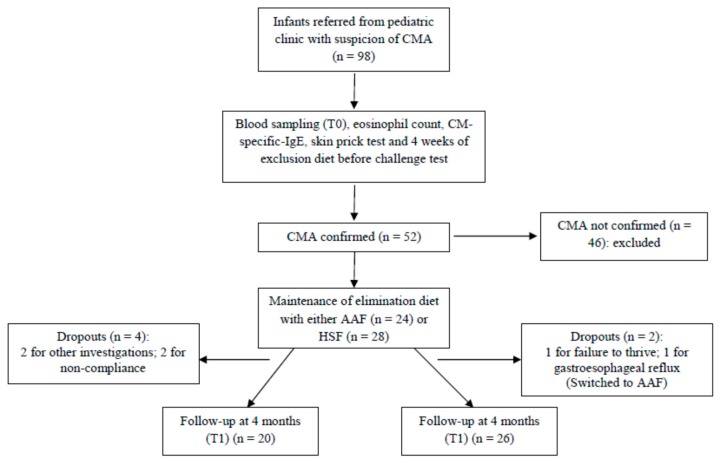
Flow of participant progress through the study.

The study received ethical approval from the Faculty of Medicine of ABC (approval number 292/2009). Infants were recruited from a gastroenterology and pediatric allergy clinic at the Faculty of Medicine of ABC between March 2010 and June 2011. Infants suspected of CMA were screened, by measuring total and CM-specific IgE and blood eosinophil numbers, skin prick testing, and exclusion of milk and dairy products from the diet for 4 weeks; if this was associated with an absence of clinical symptoms, it was followed by an open challenge test. Ninety-eight infants were screened and CMA was confirmed in 52 (Figure 1). In addition to confirmed CMA, included infants had to have been born at a gestational age of between 37 to 42 weeks, to have had a birth weight between 2500 and 4000 g, and to not have any malformation, congenital heart disease, metabolic disease, or kidney, liver or central nervous system diseases. The 52 in whom CMA was confirmed were enrolled into a prospective, randomized and controlled study (Figure 1). Informed written consent was obtained from the parents of these infants. The infants with CMA were treated with an elimination diet for 120 days, by replacing CM with a hydrolyzed soy protein formula (HSF; Alergomed, ComidaMed) (*n* = 28) or a free amino acid formula (AAF; AminoMed, ComidaMed) (*n* = 24). Alternate infants were assigned to HSF or AAF. Blood samples were collected before treatment, during active disease (T0) and after 120 days, when symptoms had disappeared (T1). During the 120-day study period, six infants dropped out (Figure 1). An additional 11 healthy infants who had received exclusive breast feeding up to 4 months of age were included as a comparator group; they provided a single blood sample.

### 2.2. Open Oral Challenge Test with Cows’ Milk

After 4 weeks of a diet excluding cows’ milk and its derivatives, and the discontinuation of medications—such as antihistamines, histamine H2 receptor antagonists, antidepressants, corticosteroids, anti-leukotrienes, bronchodilators, brometro ipratropium, theophylline and cromolyn—which could affect the outcome, an open oral challenge test with CM was used to confirm a CMA diagnosis. The test was conducted in a safe environment, with equipment for emergency treatment, which ensured no contact with patients with infectious diseases, and provided clean and comfortable conditions suitable for a long stay [18,19]. Using a polymeric formula with a low lactose content and intervals between each dose of 15 to 20 minutes, the test was started, using skin contact with gauze soaked in CM formula. After the first intervals, this was followed with oral mucosa contact and ingestion of a volume of 1 to 3 mL of CM formula at each interval, progressing to a final total volume of 100 mL of formula. During the test, vital signs were monitored and a detailed physical examination was performed, before administering the next dose. The test was discontinued immediately if signs or symptoms characteristic of allergy to CM appeared, and antihistamine or epinephrine was administered. The clinical characteristics considered to indicate IgE-mediated CMA were rash, hives, angioedema, hyperemia, pruritus (skin, lips, mouth and throat, nasal or eye), runny nose, sneezing, coughing, wheezing, watery eyes, nausea and vomiting [20,21], and those to indicate non-IgE mediated CMA were nausea, vomiting, diarrhea, abdominal distension and rectal bleeding. The test was considered positive when more than one event was observed, involving one or more systems. In young children, especially those who could not speak, responses such as putting hands in the mouth, tongue itch, scratching of the neck or behaviour change were considered evidence of a significant reaction [22]. Patients were followed by the attending physician until the end of the procedure (2 h after the last dose of the CM) and re-evaluated by the same physician after 24 h and 4 weeks.

### 2.3. Blood Tests

Blood tests performed included measurement of circulating eosinophil numbers, and measurement of serum total IgE and CM-specific IgE concentrations (which were measured using UniCAP-100E; Pharmacia and Upjohn Diagnostics AB, Uppsala, Sweden). Serum CM-specific IgE levels of less than 0.35 IU/mL were classified as undetectable.

### 2.4. Skin Prick Tests

Skin prick tests were conducted on the patient’s left forearm using commercial allergen extracts (Bencard, Brentford, UK). Histamine hydrochloride (1 mg/mL) (Bencard) was used as a positive control and physiological saline was used as a negative control. Reactions were read after 15 minutes and classified as negative if there was no reaction or if the wheal diameter was <3 mm, or positive if the wheal diameter was >3 mm.

### 2.5. Plasma Cytokine Concentrations 

Cytokine concentrations were measured by flow cytometry in plasma samples collected at T0 and T1. A human Th1/Th2/Th9/Th17/Th22 13-plex kit, capable of measuring IL-1β, IL-2, IL-4, IL-5, IL-9, IL-12p70, IL-13, IL-17A, IL-22, TNF-α and interferon (IFN)-γ (eBioscience, Hatfield, UK) was used. Assays were performed according to the manufacturer’s instructions. Data were collected on a FACSCalibur flow cytometer (BD Biosciences, Oxford, UK). Limits of detection (pg/mL) were IL-1β (4.2), IL-2 (16.4), IL-4 (20.8), IL-5 (1.6), IL-9 (1.5), IL-10 (1.9), IL-12p70 (1.5), IL-13 (4.5), IL-17A (2.5), IL-22 (43.3), IFN-γ (1.6) and TNF-α (3.2). Values below the limit of detection were set at half of this.

### 2.6. Statistical Analysis

Data were checked for normality using Kolmogorov–Smirnov and D’Algostino and Pearson omnibus normality tests. Cytokine concentrations were not normally distributed. Therefore, they are presented as the median and 10th and 90th percentiles. Comparisons between groups were made using an unpaired *t*-test, the Mann–Whitney test or the Chi-squared test, depending upon the nature of the data. Comparisons within a group, between T0 and T1, were made using the Wilcoxon rank test. Correlations were assessed using Spearman’s test. In all cases, the level of statistical significance was set at *p* < 0.05.

## 3. Results

### 3.1. Study Population

The characteristics of the infants studied are shown in Table 1. All infants were Caucasian. There were no differences between HSF and AAF groups for any of these characteristics. However, infants with CMA were more likely to have been born by Cesarean section, to have better educated mothers, and to have been breastfed for a shorter duration than the healthy breastfed (BF) comparator group (Table 1).

Clinical symptoms were classified as gastrointestinal, respiratory, skin or systemic at T0 and are reported in Table 2. The number of patients was not enough for statistical stratification according to these symptoms. Table 3 shows the blood eosinophil numbers and the response to the ImmunoCap test (total and CM-specific IgE) at T0. There were no significant differences in these parameters between infants assigned to HSF or AAF (data not shown). Infants with CMA had higher plasma concentrations of IL-4 and IL-13 and lower concentrations of IL-9, IL-17A and IFN-γ at T0 than BF infants (Table 4). Plasma cytokine concentrations at T0 did not differ according to the type of allergy (IgE or non-IgE mediated) (data not shown) and did not differ between infants with CMA assigned to HSF or AAF (data not shown). IL-5 was detected in only seven (15%) infants with CMA at T0. At T0, there were significant positive correlations between blood eosinophil numbers and plasma concentrations of IL-4 (*r* = 0.510; *p* < 0.001), IL-9 (*r* = 0.316; *p* < 0.001), IL-17A (*r* = 0.346; *p* < 0.001) and IL-22 (*r* = 0.174; *p* = 0.012). There were no significant correlations between plasma cytokine concentrations and concentrations of either total or CM-specific IgE (data not shown).

### 3.2. Effect of Dietary Treatment on Plasma Cytokine Concentrations 

CM protein exclusion using either HSF or AAF for 120 days resulted in alleviation of clinical symptoms. This was associated with decreased concentrations of IL-4, IL-13 and IL-22, irrespective of whether HSF or AAF was used (Table 4). CM protein exclusion was associated with increased IL-17A (Table 4). There was no significant effect of treatment on the concentrations of the other cytokines analyzed (IL-1β, IL-2, IL-5, IL-12p70, TNF-α and IFN-γ). At the end of treatment (i.e., T1), there were no differences in cytokine concentrations between the HSF, AAF and BF groups.

## 4. Discussion

Of 98 infants referred from a pediatric clinic with suspected CMA, 52 had the diagnosis confirmed. A CM elimination diet using either HSF or AAF was able to alleviate clinical symptoms after 120 days. Consistent with the involvement of Th2 cells in the pathogenesis of atopic disease [5,6,23], we found plasma concentrations of IL-4 and IL-13 to be elevated in infants with CMA compared with the healthy BF comparator group. Surprisingly, IL-5 concentration was below the limit of detection in most infants with CMA in the current study. This is in contrast to the finding of high serum IL-5 levels—which are associated with eosinophilia in infants with CMA—in an earlier study, although after 2 weeks on a CM elimination diet, IL-5 became undetectable in that study [24]. One reason why the findings for IL-5 differ between the current study and that of Matsumoto et al. [24] may be because the extent of eosinophilia was much less in the current study, indicating less severe disease. In addition to the elevated Th2 cytokines, in the current study, plasma concentrations of IL-9 and IL-17A were lower in infants with CMA than in the comparator BF group. However, both of these cytokines, and IL-22, were linearly positively associated with the extent of eosinophilia. This latter observation suggests roles for IL-9, IL-17A and IL-22 in CMA that are consistent with current thinking [7,8,12,13,14]. 

IL-22 can promote pathological airway inflammation and can cause barrier damage, although IL-22 can also promote epithelial cell proliferation and repair of the skin, airway or intestines [25,26,27]. The positive correlation of IL-22 concentration with eosinophilia in the current study, and the decrease in IL-22 with CM elimination, suggest a role for this cytokine in the on-going pro-inflammatory state that exists in CMA. Although the IL-22 concentration was not significantly elevated in infants with CMA compared with healthy BF infants, it was, on average, numerically higher. Th9 cells are now recognized as an important factor in allergic airway disease [8,15,16,28]. Lung selective expression of IL-9 can promote airway inflammation due to eosinophil and lymphocyte infiltration, epithelial cell hypertrophy, mucus production and deposition of collagen [28]. Surprisingly, we found lower IL-9 concentrations in the plasma of infants with CMA at T0, and we also saw a very small, but significant, reduction in IL-9 when clinical symptoms were abolished (i.e., at T1). This latter observation indicates that IL-9 may play a role in maintenance of the allergic condition. Furthermore, the IL-9 concentration correlated positively with eosinophilia. Thus, the elevation of cytokines—indicating activity of Th2, but also of Th9 and Th22 cells in infants with CMA—suggests that each of these cell types is involved in CMA, while the known roles of the cytokines suggest synergistic actions in the pathogenesis of CMA (symptom exacerbation, eosinophilia and tissue inflammation). 

Tissue-infiltrating Th17 cells have been described as important factors in several chronic inflammatory conditions, such as psoriasis, rheumatoid arthritis, multiple sclerosis, inflammatory bowel disease, allergic asthma and atopic eczema [12,13]. The lower concentration of IL-17A in the plasma of infants with CMA at T0, and its elevation when clinical symptoms were abolished (i.e., at T1), question the role that IL-17 plays in allergic conditions, specifically in the tolerance to CM proteins.

Infants with CMA had a lower plasma concentration of IFN-γ than healthy BF infants in T0. IFN-γ is a representative Th1 cytokine, and the lowered concentration supports the idea of an imbalance of Th cells, away from Th1 and towards Th2, in allergic conditions [5,8,29]. IFN-γ and the Th1/Th2 balance are targets for treating allergic conditions [29,30]. In the current study, both HSF and AAF shifted this balance away from Th2 and towards Th1.

Both dietary treatments used (HSF and AAF) had equal efficacy in alleviating clinical symptoms of CMA and altering plasma cytokine concentrations. Two infants who started in the HSF group—one with failure to thrive and the other with gastroesophageal reflux—had to switch to the AAF group due to non-remission of symptoms. It is also of interest that, of the 98 infants referred from the pediatric clinic with suspicion of CMA, only 52 were confirmed after a strict diagnostic protocol. This suggests that a number of infants who do not have CMA may be being treated as if they do. Both a proper diagnosis of CMA, and the use of an extensively hydrolysed formula as the first-choice treatment, should be encouraged.

In the current study, infants with CMA were more likely to have been born by Cesarean section than those in the healthy BF group. Some previous studies have reported that Cesarean delivery results in different gut microbiota compared with vaginal delivery [31,32,33] and that this different microbiota prolongs immunological immaturity and increases the risk of development of allergic disease [34,35,36,37]. The infants presenting with CMA also had a shorter period of breastfeeding than seen in the healthy BF group. Both the shorter duration of breast feeding and the early introduction of cows’ milk protein could be related to development of CMA.

The strengths of this study are the strict diagnostic protocol followed, the alleviation of symptoms seen with HSF and AAF, and the determination of multiple plasma cytokines. However, the study has some limitations. First, the number of infants studied was small. This is important because the heterogeneous nature of CMA means that different phenotypes may exhibit different cytokine patterns and different responses to treatment. Secondly, the cytokines reported are circulating in plasma and we have no information as to the cellular sources of those cytokines or their tissue location. Thirdly, the infants in the comparator healthy BF group provided only a single blood sample when they were about 12 months old, while infants with CMA provided blood samples at two ages.

## 5. Conclusions

In conclusion, infants with CMA have elevated plasma concentrations of IL-4 and IL-13. Concentrations of IL-4, IL-9, IL-17A and IL-22 are positively associated with blood eosinophil numbers in infants with CMA. Treatment of infants with CMA, with an elimination diet, decreased plasma concentrations of IL-4, IL-13 and IL-22. These data confirm a key role for the Th2 response in allergic disease and provide evidence for the involvement of Th9 and Th22 cells in allergic disease. These cells may represent important targets for future intervention.

## Figures and Tables

**Table 1:** Characteristics of infants in the hydrolyzed soy formula (HSF) and free amino acids formula (AAF) groups and of the healthy breastfed infants (BF).

Characteristic	HSF	AAF	BF
Number	26	20	11
Sex:			
Female, *n* (%)	9 (36)	11 (52)	5 (45)
Male, *n* (%)	17 (64)	9 (48)	6 (55)
Mean age at T0 (months)	8.0 ± 0.8	5.6 ± 0.6	-
(range)	2–17	2–13	
Mean age at T1 (months)	12.0 ± 0.8	9.4 ± 0.6	12.5 ± 1.8
(range)	6–21	6–17	8–25
Mode of birth:			
Cesarean, *n* (%)	23 (89) **	19 (97) **	3 (27)
Vaginal, *n* (%)	3 (11) **	1 (3) **	8 (73)
Birth weight (g)	3122 ± 56	3115 ± 41	3158 ± 88
Length at birth (cm)	47.9 ± 0.4	47.9 ± 0.9	47.8 ± 0.6
Exclusive breastfeeding (months)	2.7 ± 0.5 *	2.4 ± 0.4 *	4.9 ± 0.3
Maternal age (years)	29.3 ± 1.5	30.5 ± 1.7	26.3 ± 0.9
Maternal education:			
High school, *n* (%)	8 (30) **	8 (40) **	100 (11)
College, *n* (%)	19 (70) **	12 (60) **	0 (0)

* Different from the BF group according to an unpaired *t*-test (*p* = 0.003); ** Different from the BF group according to Chi-square (*p* < 0.001).

**Table 2:** Clinical description of infants in the hydrolyzed soy formula (HSF) and free amino acids formula (AAF) groups at T0. Data are *n* (%).

Clinical Description	HSF (*n* = 26)	AAF (*n* = 20)
Type of allergy:		
Immunoglobulin (IgE) mediated	7 (27)	3 (15)
Non-IgE mediated	15 (58)	16 (80)
Mixed	4 (15)	1 (5)
Clinical symptoms:		
Gastrointestinal		
Diarrhea/constipation/colic	11 (42)	5 (20)
Vomiting/regurgitation/reflux	7 (27)	5 (25)
Colitis/blood in stools	13 (50)	13 (65)
Respiratory		
Wheeze	6 (23)	1 (5)
Skin		
Contact urticaria	11 (42)	4 (20)
Atopic dermatitis	0 (0)	1 (5)
Systemic		
Anaphylaxis	1 (4)	1 (5)
Failure to thrive	0 (0)	4 (20)

**Table 3:** Blood eosinophil numbers and serum total, cows’ milk protein-specific and soya-specific IgE concentrations at the time of clinical evaluation (T0) in infants with CMA.

Marker of Allergy	Infants with CMA (*n* = 46)
ImmunoCap negative, % (n)	39 (18)
Blood eosinophils (cells/mm^3^) *	232.3 (72.0–693.9)
Total IgE (IU/mL) *	8.55 (2.3–195.0)
Cows’ milk protein-specific IgE (IU/mL) *	1.94 (0.04–77.64)
Anti-alpha lactalbumin IgE (IU/mL) *	1.47 (0.08–35.08)
Anti-beta lactalbumin IgE (IU/mL) *	1.38 (2.98–25.0)
Anti-casein IgE (IU/mL) *	1.12 (0.002–39.36)
Anti-soya IgE (IU/mL) *	0.0 (0.0–3.52)

* Data are shown as median (10th–90th percentile).

**Table 4:** Plasma cytokine concentrations (pg/mL) in infants with CMA before treatment (T0), and after 4 months of treatment with either AAF or HSF (T1) and in healthy breast-fed (BF) infants.

Cytokine	CMA (T0)	CMA (T1)	*P* for CMA (T0) vs. CMA (T1)	BF	*P* for CMAT0 vs. BF *
IL-1β	2.0 (2.0–48.0)	2.0 (2.0–15.5)	0.132	2.0 (2.0–13.3)	0.324
IL-2	10.0 (10.0–71.56)	39.9 (10.0–65.6)	0.948	17.8 (7.7–92.9)	0.498
IL-4	35.9 (10.0–159.9)	10.0 (10.0–60.9)	0.001	10.0 (8.7–55.6)	0.022
IL-9	1.0 (1.0–20.3)	1.0 (1.0–7.5)	0.003	2.7 (1.2–5.8)	0.038
IL-12p70	1.0 (1.0–19.1)	1.0 (1.0–8.7)	0.083	1.0 (1.0–14.3)	0.905
IL-13	48.4 (3.0–58.7)	13.0 (3.0–55.5)	0.001	3.8 (2.2–20.2)	<0.01
IL-17A	1.50 (1.50–84.5)	10.5 (1.5–40.6)	0.002	6.9 (1.0–21.3)	0.039
IL-22	145.7 (20.0–278.5)	103.2 (20.0–176.8)	0.018	109.5 (20.0–166.8)	0.128
TNF-α	4.9 (1.4–58.8)	3.4 (1.0–46.7)	0.247	8.1 (1.3–29.8)	0.229
IFN-γ	1.0 (1.0–45.9)	1.0 (1.0–85.6)	0.138	5.8 (1.0–81.9)	0.033

Data are shown as median (10th–90th percentile); * Mann–Whitney test.

## Abbreviations

The following abbreviations are used in this manuscript:

AAF	free amino acid formula
BF	breast fed
CM	cows’ milk
CMA	cows’ milk allergy
HSF	hydrolysed soy protein formula
IFN	interferon
IgE	immunoglobulin E
IL	interleukin
SPT	skin prick test
TNF	tumor necrosis factor
